# Circulating glucose levels inversely correlate with *Drosophila* larval feeding through insulin signaling and SLC5A11

**DOI:** 10.1038/s42003-018-0109-4

**Published:** 2018-08-13

**Authors:** Rupali Ugrankar, Pano Theodoropoulos, Fatih Akdemir, W. Mike Henne, Jonathan M. Graff

**Affiliations:** 1 Department of Developmental Biology, University of Texas Southwestern Medical Center, 5323 Harry Hines Blvd., Dallas, TX 75390 USA; 2 Department of Cell Biology, University of Texas Southwestern Medical Center, 5323 Harry Hines Blvd., Dallas, TX 75390 USA; 3 Department of Basic Sciences, Medical Biology, Ataturk University, 25240 Erzurum, Turkey; 4 Department of Molecular Biology, University of Texas Southwestern Medical Center, 5323 Harry Hines Blvd., Dallas, TX 75390 USA; 5 Department of Internal Medicine, Division of Endocrinology, University of Texas Southwestern Medical Center, 5323 Harry Hines Blvd., Dallas, TX 75390 USA

## Abstract

In mammals, blood glucose levels likely play a role in appetite regulation yet the mechanisms underlying this phenomenon remain opaque. Mechanisms can often be explored from *Drosophila* genetic approaches. To determine if circulating sugars might be involved in *Drosophila* feeding behaviors, we scored hemolymph glucose and trehalose, and food ingestion in larvae subjected to various diets, genetic mutations, or RNAi. We found that larvae with glucose elevations, hyperglycemia, have an aversion to feeding; however, trehalose levels do not track with feeding behavior. We further discovered that insulins and SLC5A11 may participate in glucose-regulated feeding. To see if food aversion might be an appropriate screening method for hyperglycemia candidates, we developed a food aversion screen to score larvae with abnormal feeding for glucose. We found that many feeding defective larvae have glucose elevations. These findings highlight intriguing roles for glucose in fly biology as a potential cue and regulator of appetite.

## Abstract (web summary)

Rupali Ugrankar et al. show that *Drosophila* larvae with high levels of circulating glucose, but not trehalose, don’t eat much. This study suggests that circulating glucose communicates with insulin signaling and the sodium/solute co-transporter SLC5A11 in the brain to suppress larval appetite.

## Introduction

In the past few decades, there has been a steady rise in the worldwide incidence of type 2 diabetes. This increase has been linked to greater energy intake relative to output, due to increased food consumption, and subsequent weight gain and obesity^1–6^. In mammals, food consumption is regulated by a variety of inputs, one of which appears to be blood glucose levels^7,8^. In 1955, Jean Mayer proposed a glucostatic theory in which transient decreases and increases in blood glucose concentrations perceived by specialized “glucoreceptors” on hypothalamic cells trigger perceptions of hunger and satiety, respectively^7,8^. A more recent study observed that small declines in blood glucose levels often preceded spontaneous meal initiation in rats^9^. Thus, at least in principle, depletion of fuel substrates should trigger initiation of feeding while repletion should cause cessation of feeding^7,10^. This notion is also well illustrated in people with insulin secreting tumors^11^.

The regulation of appetite and glycemia is likely the outcome of a complex interplay of multiple metabolic, hormonal, and neural signals that are not yet completely elucidated^10,12,13^. Studies in mice and rats have uncovered numerous anorexigenic “satiety factors”, for example, cholecystokinin, glucagon, and leptin, while orexigenic factors, such as ghrelin, stimulate food intake via regulation of the Neuropeptide Y axis^10,14,15^. As in mammals, neural signals have been implicated in the control of fly feeding behaviors. For example, activation of insulin signaling in the nervous system suppresses larval feeding and overexpression of Neuropeptide F, the fly homolog of mammalian neuropeptide Y, promotes feeding, even of noxious or unfamiliar foods^16–19^. Other fly neuropeptides linked to the regulation of food intake include short neuropeptide F and CCHAmide-2, and drosulfakinin and hugin, which increase or decrease larval feeding, respectively^20–23^.

To attempt to explore a potential relationship between circulating carbohydrates and feeding behavior, we probed mid-third instar larvae, as their incessant feeding at a consistent rate provides a potentially stable state. Feeding larvae maintain glucose levels between 6–9 mg/dl and trehalose levels at ≥ 1500 mg/dL^24^, hinting at a highly efficient carbohydrate regulatory mechanistic. We found that increasing the concentration of dietary sucrose was associated with increased levels of circulating glucose and trehalose but decreased rates of feeding in larvae. Further, we observed that feeding aversion appeared to correlate with increasing glucose, but not trehalose, levels. Moreover, severe hyperglycemia also delayed larval foraging activity, indicative of enhanced satiety. Alleviation of hyperglycemia by fasting or through pharmacological means rescued the feeding defect in “flyabetic” larvae. Further, insulin signaling was increased in food averse hyperglycemic larvae. SLC5A11, a sodium/solute co-transporter-like neuronal protein showed decreased expression, and appears to be regulated by insulin activity. Microarray analyses of flyabetic larvae further validated the roles of insulin signaling and SLC5A11 in glucose sensing, and identified other potential candidates. Finally, we evaluated feeding behavior of >2000 P-element lines and identified 63 ‘satiety’ mutants that displayed a very marked aversion to food; ~50% of these were hyperglycemic, supporting the notion that food-averse mutants are an enriched pool to identify genes that regulate glucose levels. Taken together, our data support the notion that there is a strong correlation between hemolymph glucose levels and *Drosophila* feeding behavior, and that glucose may act as a cue to modulate feeding behavior.

## Results

### Hemolymph carbohydrates may modulate larval feeding behavior

**Fig. 1:**
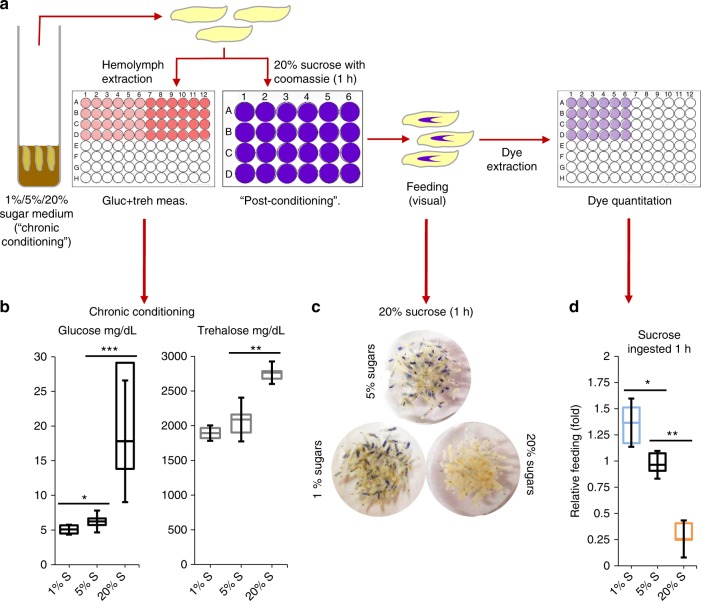
Changes in hemolymph carbohydrates appears to influence sucrose ingestion by larvae. **a** Larvae chronically reared on media containing different concentrations of sugars were subjected to glucose, trehalose, and post-conditioning feeding assays. **b** Circulating glucose (left) and trehalose (right) levels were measured in mid-third instar *w*^*1118*^ larvae cultured in media containing 1%, 5% or 20% sugars. *n* ≥ 4 each (≥10 larvae per replicate). **c**, **d** Relative dye ingested in 1 h of post-conditioning 20% sucrose feeding was observed (**c**) and measured (**d**) in *w*^*1118*^ larvae cultured on 1%, 5%, or 20% sugars. ≥ 50 larvae per treatment. For dye quantification, *n* ≥ 3 (20 larvae per replicate). Error bars indicate standard deviation (s.d.). Statistical significance was assessed by two-tailed Student’s *t*-test, *P < 0.05, **P < 0.01, ***P < 0.001

We previously noticed during RNAi screening for glucose regulatory genes^24^ that hyperglycemic larvae appeared to have a delayed interest in feeding when transferred to fresh media. To attempt to investigate a potential connection between circulating carbohydrates and food consumption, we reared (“chronic conditioning”) mid-third instar *w*^*1118*^ larvae on 1%, 5% (standard food), and 20% sugar media. We then measured the levels of glucose and trehalose in *w*^*1118*^ larval hemolymph (Fig. 1a), and found that, relative to larvae fed 5% sugars, the 1% sugar-fed larvae had slightly reduced glucose (0.8 fold), but not trehalose, levels, and that 20% sugar feeding produced both hyperglycemia (3.3 fold) and hypertrehalosemia (1.4 fold) (Fig. 1b). We then transferred larvae from the 1%, 5%, and 20% sugar media to 1 h of post-conditioning culture in a 20% sucrose solution supplemented with Coomassie dye, to allow us to visualize and to quantitate food consumption (Fig. 1a). The larvae reared on 1% and 5% sugar media had roughly equal gut dye intensity in the visual assay (Fig. 1c). However, the 20% sugar-fed cohorts had visibly less food in their gut after 1 h of post-conditioning dye feeding (Fig. 1c). To more precisely measure food consumption, we extracted Coomassie from larvae and quantitated dye levels using a spectrometric assay, which showed that 1% sugar-reared *w*^*1118*^ larvae ingested 32% more sucrose, while the 20% sugar-reared larvae reduced sucrose intake by 65%, relative to 5% sugar-reared euglycemic counterparts (Fig. 1d). These data indicate a potential link between hemolymph carbohydrates and feeding. Further, the observations that the 1% cohorts displayed increased food intake and low glucose levels, but normal trehalose levels hint at the possibility that hemolymph glucose, rather than trehalose, might be the carbohydrate link to feeding behavior control.

**Fig. 2:**
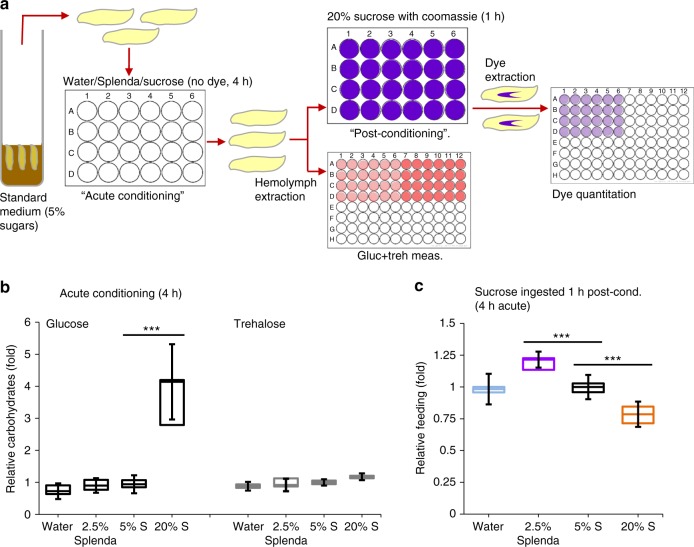
Glucose levels but not trehalose levels may be linked to altered feeding by larvae. **a** *w*^*1118*^ controls reared in standard media were provided water, 2.5% Splenda, 5% sucrose, or 20% sucrose and after 4 h of acute conditioning carbohydrate levels and dye ingested in 1 h of post-conditioning feeding were measured. **b** Relative glucose (left) and trehalose (right) levels were measured in *w*^*1118*^ larvae after 4 h conditioning on different media. *n* ≥ 4 each (≥10 larvae per replicate). **c** Quantification of post-conditioning feeding (1 h) in larvae. n ≥ 8 each (20 larvae per replicate). Error bars indicate s.d. Statistical significance was assessed by two-tailed Student’s *t*-test, *P < 0.05, **P < 0.01, ***P < 0.001

We then measured feeding in another setting where glucose, but not trehalose, levels are changed: larvae subjected to acute feeding of concentrated sucrose respond by increasing hemolymph glucose levels, but they do not alter trehalose levels^24,25^. Therefore, we transferred *w*^*1118*^ larvae reared on standard food to 4 h of “acute” conditioning in water, 5% sucrose, 20% sucrose, or 2.5% Splenda i.e. sucralose (equivalent sweetness to 20% sucrose, negligible calories) solutions (without dye). After 4 h, half of the larvae from each of the four groups were analyzed for post-conditioning carbohydrate levels, while the others were transferred to a 1 h incubation in Coomassie dye-supplemented 20% sucrose solution to assess post-conditioning food intake (Fig. 2a). All the larvae had equal trehalose levels, while the larvae acutely conditioned on 20% sucrose for 4 h had significantly increased glucose levels by 3.6-fold (Fig. 2b). These hyperglycemic larvae also had significantly reduced food ingestion by 22%, relative to the euglycemic larvae conditioned on water, Splenda, or 5% sucrose (Fig. 2c), supporting a potential association between hyperglycemia and decreased feeding in high sucrose-conditioned *w*^*1118*^ larvae. The larvae fed 2.5% Splenda ate slightly more (1.8-fold) of the post-conditioning media despite no significant decrease in glycemia (Fig. 2b, c). It appears that larvae may simply prefer the taste of Splenda, as *w*^*1118*^ larvae consumed the same amount of water, 5% sucrose, and 20% sucrose but ingested increased amounts (1.6 fold) of 2.5% Splenda in the first hour of conditioning (Supplementary Fig. 1b). Taken together, these data support the notion that circulating glucose, but not trehalose, levels appear to be one of the regulators of larval feeding behavior.

### Elevations in glucose correlate with reduced feeding

**Fig. 3:**
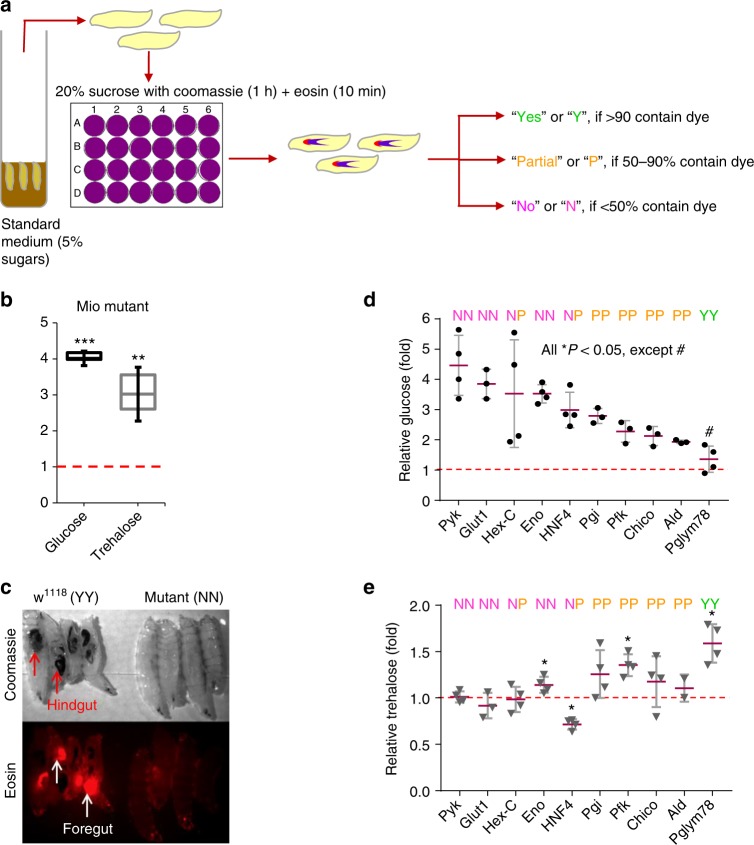
Metabolic mutants display reduced sucrose feeding. **a** Schematic of the qualitative dual-dye liquid feeding assay. Larvae were scored for dye ingestion, relative to controls, after 1 h on 20% sucrose supplemented with Coomassie and Eosin dyes. Eosin dye was added at 50 min. **b** Hyperglycemia and hypertrehalosemia detected in *Mio* mutant larvae. **c** Microscopic image of Coomassie blue and Eosin dyes in *w*^*1118*^ control and *Mio* mutant guts after 1 h culture in 20% sucrose. **d**, **e** Glucose (**d**), trehalose (**e**), and two-dye sucrose feeding (**d**, **e**) were scored for key proof-of-principle mutants (insulin pathway- *Glut1, Chico; MODY- Hex-C, HNF4*; Glycolysis- *Pyk, Eno, Pgi, Pfk, Ald, Pglym78*). *n* = 4 each (≥10 larvae per glucose/trehalose replicate). ≥30 larvae per feeding sample. Error bars indicate s.d. Statistical significance was assessed by two-tailed Student’s *t*-test, *P < 0.05, **P < 0.01, ***P < 0.001

To attempt to further discriminate whether the glucose and/or trehalose fraction might regulate larval feeding, we probed collections of P-element metabolic mutants^24^. For these larger-scale efforts, we developed a qualitative dual-dye visual liquid feeding assay amenable to high throughput analyses (Fig. 3a). In this assay, we cultured control and mutant larvae on standard food and then transferred the larvae to a 1 h conditioning in 20% sucrose solution containing Coomassie blue (stains hind-gut) and fluorescent Eosin (stains fore-gut). After the hour, we scored the larvae for food consumption (Fig. 3a), and we quantitated carbohydrate levels. In this assay, the hyperglycemic, hypertrehalosemic P-element mutant for *Mio*, fly homolog of mammalian *ChREBP*, larvae demonstrated a strong feeding aversion and an NN (N for Coomassie, N for Eosin) phenotype, as illustrated by the absence of both dyes in their intestine (Fig. 3b, c). Mutants for insulin pathway-related (*Chico, Glut1*), MODY (maturity onset diabetes of the young) homologs (*HNF4, Hex*), and glycolysis genes (*Hex, Pgi, Pfk, Ald, Pglym78, Eno*, and *Pyk*) showed increased glucose levels relative to controls, ranging from 1.5-fold to 4.5-fold, but variable trehalose levels from low to high (0.7-fold to 1.6-fold) (Fig. 3d, e). In these studies, food consumption inversely correlated with glucose levels, but did not track with trehalose levels (Fig. 3d, e). These results, using a visual food aversion assay, were similar to our findings with the quantitative assay.

**Fig. 4:**
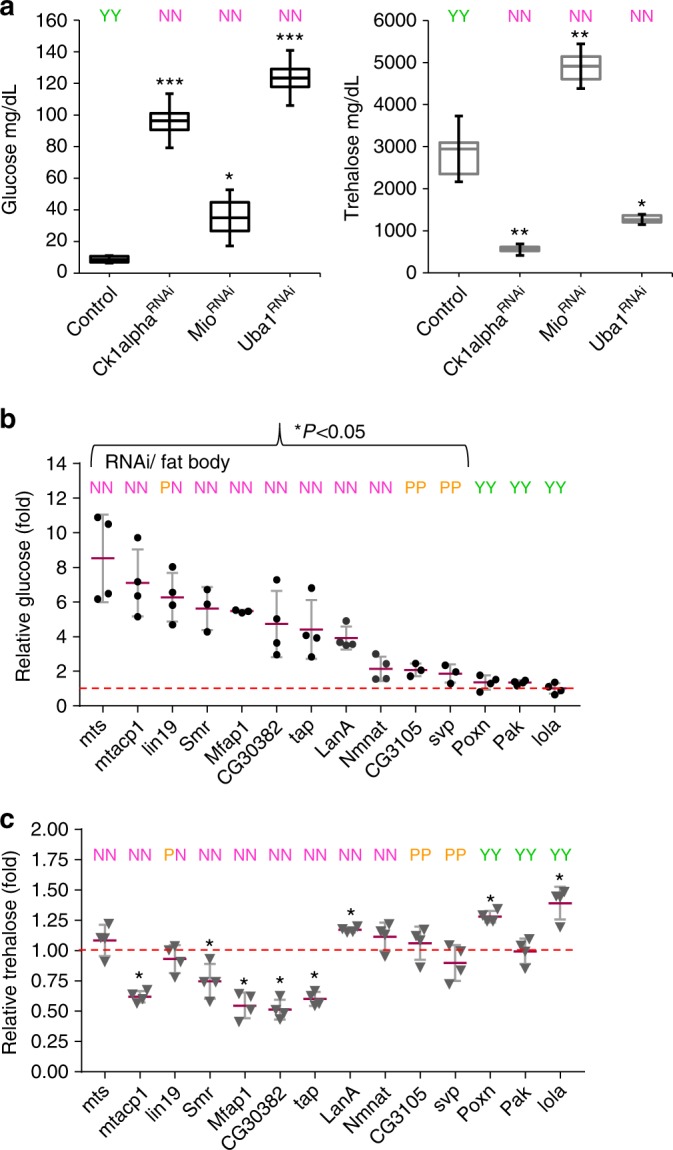
Hemolymph glucose elevations induce satiety behaviors. **a** Glucose (left) and trehalose (right) levels, and two-dye sucrose feeding was measured in *w*^*1118*^*; Dcg-Gal4* control and “flyabetic” larvae; dotted line indicates control concentrations. **b**, **c** Glucose (**b**), trehalose (**c**)^24^, and two-dye sucrose feeding (**b**, **c**) were concurrently assayed in 14 other hyperglycemia candidates (fat body knockdowns), and plotted relative to controls levels. *n* = 4 each (≥10 larvae per glucose/trehalose replicate). ≥30 larvae per feeding sample Error bars indicate s.d. Statistical significance was assessed by two-tailed Student’s *t*-test, *P < 0.05, **P < 0.01, ***P < 0.001

In our next approach, we evaluated feeding in collections of RNAi knockdown flies that we had previously shown to have altered levels of glucose and trehalose^24^. As a step, we selected three strong “flyabetes” hits *Mio*, *Ck1alpha*, *and Uba1* that we had identified in our previously reported “Glucome” screens^24^. Fat body loss-of-function of all three candidates produced extreme sucrose aversion (NN) and hyperglycemia: a 4-fold, 11-fold, and 14-fold glucose increase in *Mio*^*RNAi*^, *Ck1alpha*^*RNAi*^, *Uba1*^*RNAi*^ larvae, respectively (Fig. 4a). Yet, only *Mio*^*RNAi*^ larvae had high trehalose (1.8 fold), while, *Ck1alpha*^*RNAi*^ and *Uba1*^*RNAi*^ had reduced trehalose levels to 0.22-fold and 0.5-fold, respectively. Un-induced RNAi larvae for *Mio*, *Ck1alpha*, and *Uba1* had control levels of carbohydrates, and fed normally (Supplementary Fig. 2). These data mirror the observations with the various diets and P-element mutants.

We next simultaneously analyzed glucose, trehalose, and feeding using the dual dye assay with additional 14 flyabetes candidates, from the aforementioned RNAi screens^24^. Larvae with the highest glucose levels did not eat (N); those with intermediate glucose elevations often displayed moderate aversion (P), while candidates that were not significantly hyperglycemic in this instance fed normally (Y) (Fig. 4b, c). Remarkably, out of a total of 11 hyperglycemic food-averse candidates, 5 had normal trehalose, and 5 had lower trehalose. Although this is only an association, together these data suggest that circulating glucose, but likely not trehalose, may function as a signaling metabolite and may have important roles in appetite control.

### Hyperglycemic larvae show lower motivation to work for food

**Fig. 5:**
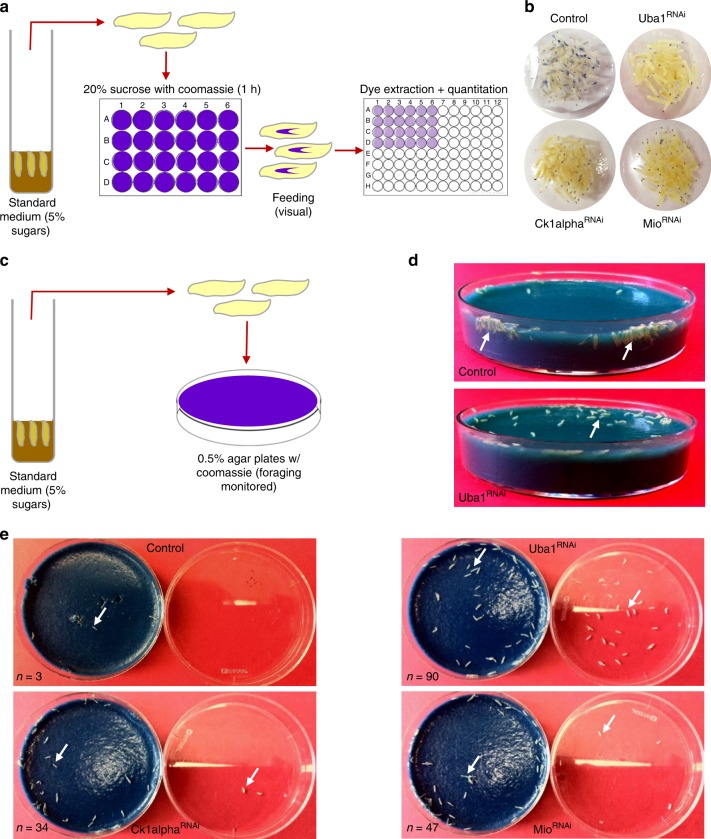
Hyperglycemic larvae have abnormal foraging behaviors. **a** *w*^*1118*^*; Dcg-Gal4* control and flyabetic larvae reared on standard food were provided Coomassie dye-supplemented 20% sucrose and after 1 h feeding was assayed. **b** Relative dye ingested in 1 h of 20% sucrose feeding was visually detected in control and flyabetic larvae. **c** *w*^*1118*^*; Dcg-Gal4* control and flyabetic larvae (*n* = 100 each) were transferred to Coomassie-dyed food plates, and initiation of foraging was monitored. **d**, **e** Burrowing (**d**) and foraging (**e**) activity of control and flyabetic larvae on 0.5% agar media was observed 2 h after transfer. 100 larvae transferred to each agar plate. Number of larvae not yet inside the food is indicated on the bottom left corner of each panel (**e**). Error bars indicate SEM. Statistical significance was assessed by two-tailed Student’s *t*-test, *P < 0.05, **P < 0.01, ***P < 0.001

Foraging (active food seeking behaviors) is another established and independent indicator of appetite^16–18^. To further evaluate the notion that glucose might control appetite, we analyzed the foraging behavior of flyabetes hits *Ck1alpha*^*RNAi*^*, Mio*^*RNAi*^, *Uba1*^*RNAi*^ (Fig. 5a, b, Supplementary Fig. 3a). We placed *RNAi*, and control (*w*^*1118*^*; Dcg-Gal4*) larvae on Coomassie-supplemented 0.5% (low) agar food plates (Fig. 5c). While most *w*^*1118*^ larvae burrowed deep inside the food and resumed feeding within the first hour, all hyperglycemic larvae lingered on the food surface and many wandered away from the food to the plate lid (Fig. 5d, e). After 3 h, larvae were analyzed for food ingestion; *Ck1alpha*^*RNAi*^*, Mio*^*RNAi*^, and *Uba1*^*RNAi*^ larvae contained ~35%, 50%, and 85% less food, respectively, relative to those of controls (Supplementary Fig. 3c).

Solid food is generally less attractive to larvae^16^ and difficulties getting to the food source may be a barrier to feeding. Therefore, we also tested foraging activity on minimally resistant 0.2% agar media and agar-free yeast-sucrose paste; however, lowering resistance did not persuade hyperglycemic larvae into the food (Supplementary Fig. 3d-e). Collectively, these data indicate that hyperglycemic larvae have reduced drive to obtain food.

### Hyperglycemic larvae display a general aversion to feeding

**Fig. 6:**
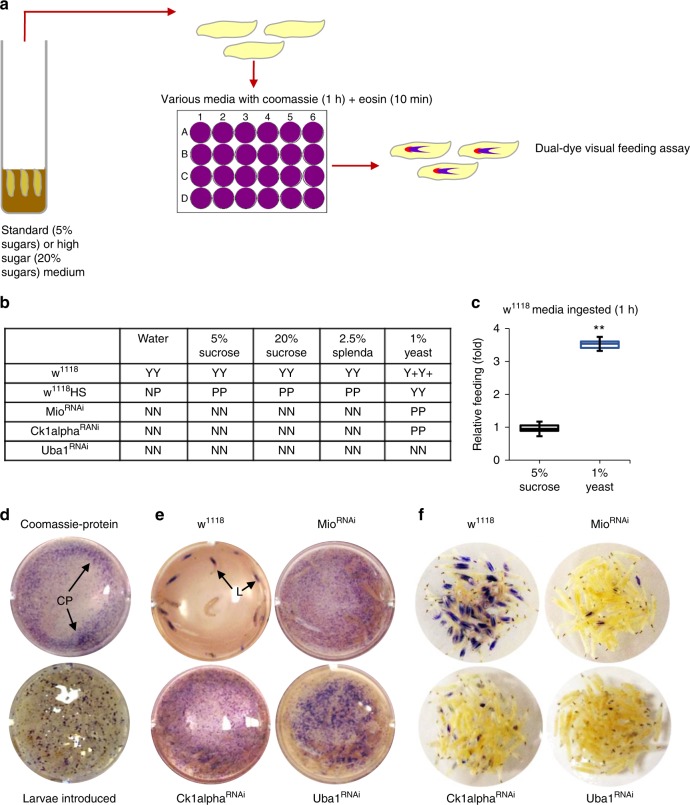
Food aversion by hyperglycemic larvae is not limited to sucrose. **a** *w*^*1118*^ control, *w*^*1118*^ high sugar-reared larvae, and flyabetic larvae (reared on standard food) were provided water, 2.5% Splenda, 5% sucrose, 20% sucrose, and 1% yeast solutions containing Coomassie and Eosin dyes for 1 h. **b** Larval guts were scored for the presence Coomassie and Eosin dyes after 1 h on various media. ≥30 larvae per treatment. **c** Relative amounts of 5% sucrose and 1% yeast ingested in 1 h by *w*^*1118*^ control larvae reared on standard food. *n* = 4 each (20 larvae per replicate). **d** *w*^*1118*^ control and flyabetic larvae were transferred to a solution containing insoluble Coomassie blue-protein aggregates (CM) and allowed to feed for 2 h. (i) Coomassie-protein aggregate solution in well, before addition of larvae (ii). Larvae just added to solution (*t* = 0). **e** Solutions were pictured 2 h later, following removal of control and flyabetic larvae. L = Larvae left behind. Un-ingested Coomassie-protein speckles are visible in solutions, post-feeding by *RNAi* larvae. **f** Larvae were separately photographed at 2 h. Error bars indicate s.d. Statistical significance was assessed by two-tailed Student’s *t*-test, *P < 0.05, **P < 0.01, ***P < 0.001

The studies described above indicate that hyperglycemic larvae have a reduced interest in eating calorific high sugar. To assess if diminished appetite was more general, we transferred hyperglycemic larvae 20% sucrose-reared *w*^*1118*^ and *Mio*^*RNAi*^*, Ck1alpha*^*RNAi*^*, Uba1*^*RNAi*^, and controls, to water, 2.5% Splenda, 5% sucrose, 20% sucrose, and soluble 1% yeast (high protein) solutions containing Coomassie and Eosin dyes, for 1 h (Fig. 6a). Controls reared on 5% sugars ate all food media, but the amount ingested was much greater in the presence of yeast (Y + Y + ) (Fig. 6b, c). *w*^*1118*^ HS and *RNAi* larvae consumed little to none of the water, Splenda, and sucrose (Fig. 6b). Of note, we sometimes observed a partial rescue of the feeding aversion in all hyperglycemic larvae, except *Uba1*^*RNAi*^, when media contained proteinaceous yeast (Fig. 6a, b), indicating that protein sensing may induce a higher drive to eat. However, *RNAi* larvae transferred to a solution of Coomassie-protein aggregates for 2 h ate almost none of the protein (Fig. 6d), in contrast to euglycemic controls that left behind a clear solution. These data imply that increases in circulating glucose induce a general rather than a carbohydrate-specific feeding suppression.

### Lowering glucose in flyabetic larvae triggers re-feeding

**Fig. 7:**
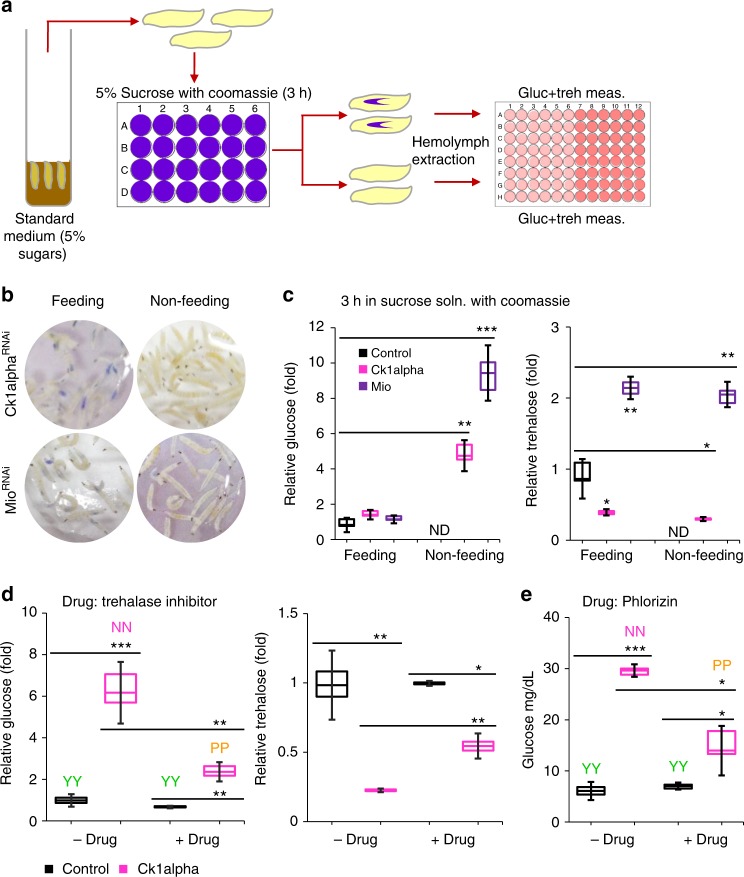
Larvae resume feeding on alleviation of hyperglycemia. **a** *w*^*1118*^*; Dcg-Gal4* control and flyabetic larvae (≥75 larvae each) were cultured in 5% sucrose solution for 3 h, after which glucose and trehalose were measured in feeding and non-feeding flyabetic larvae, and compared to controls. **b** Feeding and non-feeding flyabetic larvae imaged at 3 h. **c** Relative glucose (left) and trehalose (right) levels, compared to feeding controls, were measured in feeding and non-feeding larvae. **d** Relative glucose (left) and trehalose (right) levels, and two-dye sucrose feeding were compared in control and *Ck1alpha*^*RNAi*^ larvae chronically cultured in the presence or absence of trehalase inhibitor drug. **e** Glucose levels were measured and two-dye sucrose feeding observed in controls and *Ck1alpha*^*RNAi*^ larvae cultured in the presence or absence of Phlorizin. *n* ≥ 3 each (≥10 larvae per glucose/trehalose replicate). ≥30 larvae per feeding sample. Error bars indicate s.d. Statistical significance was assessed by two-tailed Student’s *t*-test, *P < 0.05, **P < 0.01, ***P < 0.001

Most flyabetic flies are slightly developmentally delayed but survive to adulthood. Unsurprisingly, *Ck1alpha*^*RNAi*^, *Mio*^*RNAi*^, and *Uba1*^*RNAi*^ larvae reared on Coomassie-supplemented standard food all contain gut dye but in highly variable amounts, (Supplementary Fig. 4a, b) which may be explained by intermittent rather than continuous food consumption. To explore if *RNAi* larvae resume feeding in the acute setting, and if this is accompanied by changes in circulating carbohydrates, we transferred *Ck1alpha*^*RNAi*^ and *Mio*^*RNAi*^ larvae to 5% sucrose containing Coomassie for 3 h, and then separated feeding (dye in gut) from as yet non-feeding larvae (no dye in gut) (Fig. 7a, b). Remarkably, we found that flyabetic larvae that ate had lowered their glucose levels to that of controls, but those that remained food averse, still had 5-fold- (*Ck1alpha*^*RNAi*^) and 9-fold-increased (*Mio*^*RNAi*^) glucose levels (Fig. 7c). In contrast, the trehalose status of all groups was unchanged (Fig. 7c). In a subsequent experiment, we transferred *Ck1alpha*^*RNAi*^, *Mio*^*RNAi*^, and control larvae to Coomassie-dyed 5% sucrose solution and monitored changes in glucose and percent larvae feeding between 2 h to 4 h post-transfer. In a subsequent experiment, average glucose levels of flyabetic larvae sampled between 2–4 h post-transfer to sucrose decreased with time, while the proportion of actively feeding larvae increased (Supplementary Fig. 4c). These data suggest that flyabetic larvae are a mix of glucose and feeding states.

To test if pharmacologically reducing glucose levels could alleviate feeding aversion, we reared control and *CK1alpha*^*RNAi*^ larvae in standard food containing vehicle or 25 µM of drug Deoxynojirimycin hydrochloride (Cayman Chemicals, # 10011718), which inhibits trehalase, an enzyme required for catalysis of trehalose to glucose^26–28^. We found that in *CK1alpha*^*RNAi*^ drug-treated larvae, glucose levels were reduced (7 to 3-fold) and trehalose levels increased (0.2 to 0.5-fold), and they had improved feeding relative to untreated *CK1alpha*^*RNAi*^ larvae (Fig. 7d). We also evaluated glucose and feeding changes in *Ck1alpha*^*RNAi*^ and control larvae cultured on food containing 10 mM Phlorizin, a drug known to interfere with mammalian glucose transport from the gut into the circulation, and demonstrated to be also effective in flies^28^. We observed that Phlorizin alleviated hyperglycemia, and partly rescued the feeding defect in treated *Ck1alpha*^*RNAi*^ larvae; glucose and feeding (qualitative and quantitative) was unaffected in Phlorizin-receiving controls (Fig. 7e, Supplementary Fig. 4d, e). These findings support the notion that hemolymph glucose functions as a signaling molecule and a cue in *Drosophila* larvae, and thereby controls feeding.

### Insulin signaling and SLC5A11 may regulate larval feeding

**Fig. 8:**
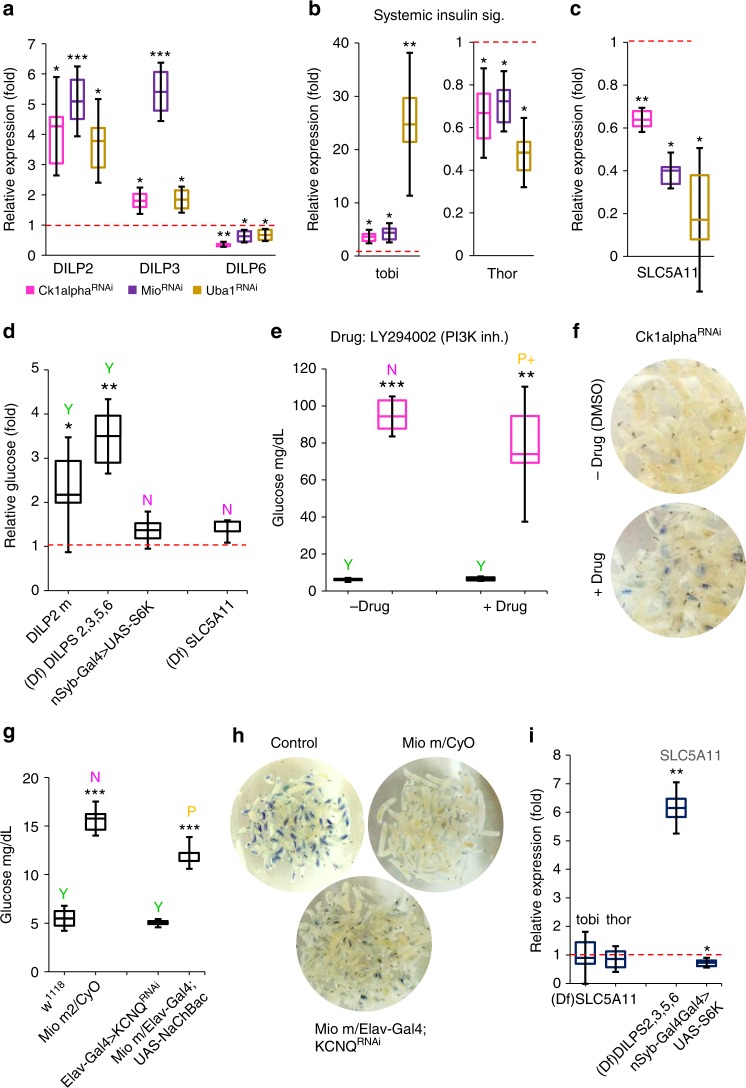
Insulin signaling and SLC5A11 (a sodium/solute co-transporter-like protein in flyabetic larvae) may be involved in glucose-responsive feeding behavior. **a** Relative levels of insulins *DILP2*, *DILP3*, and *DILP6* expressed in *Ck1alpha*^*RNAi*^*, Mio*^*RNAi*^*, and Uba1*^*RNAi*^ flyabetic larvae. *n* ≥ 3 each (20 larvae per replicate). **b** mRNA levels of insulin targets *tobi* and *Thor* in flyabetic larvae, relative to controls. **c** *SCL5A11* fold change in flyabetic larvae, relative to controls. *n* ≥ 3 each (20 larvae per replicate). **d** Relative glucose and Coomassie-dye sucrose feeding is indicated in larvae with altered insulins/ insulin signaling, and SLC5A11, relative to controls. *n* ≥ 3 each (≥10 larvae per glucose replicate). ≥30 larvae per feeding sample. **e**, **f** Glucose levels (**e**) and feeding (**e**, **f**) were assayed in *Ck1alpha*^*RNAi*^ larvae cultured in the presence or absence of PI3K inhibitor LY294002. *n* = 4 each (≥10 larvae per glucose replicate). ≥30 larvae per feeding sample. **g**, **h** Glucose (**g**) and feeding (**g**, **h**) were compared in *Mio* mutants with enhanced excitation of neuronal SLC5A11, relative to *Mio* flyabetic larvae. *n* = 4 each (≥10 larvae per glucose replicate). ≥30 larvae per feeding sample. (**i**) Relative expression of *tobi* and *Thor* in SLC5A11 loss-of-function larvae, and *SLC5A11* mRNA levels in larvae with reduced DILPS or activated insulin signaling. *n* = 3 each (20 larvae per replicate). Error bars indicate s.d. Statistical significance was assessed by two-tailed Student’s *t*-test, *P < 0.05, **P < 0.01, ***P < 0.001

Several studies have demonstrated that fly insulins are secreted in response to elevated hemolymph sugars^19,29^, and can inhibit motivated feeding in fasted larvae^16^. To test if elevations in insulin-like peptides might be part of the mechanism for blunted feeding, we measured and observed *DILP2* and *DILP3* mRNAs were increased in *Mio*^*RNAi*^,*Ck1alpha*^*RNAi*^, and *Uba1*^*RNAi*^ larvae (Fig. 8a); interestingly, in the latter two, total circulating sugars (glucose plus trehalose) are lower than controls, indicating that glucose elevations alone are sufficient to promote insulin expression. Further, the expression of *DILP6*, a fat-body derived peptide known to negatively modulate IPC insulin expression^30–32^ was lower in all three flyabetic larvae (Fig. 8a). To evaluate if and how systemic insulin signaling may be affected in flyabetic larvae, we detected transcripts of *tobi* (*target of brain insulin*) and *Thor* (*4EBP*), which are repressed and induced, respectively, by active peripheral insulin signaling^30,33^. We found that flyabetic larvae had increased *tobi* but decreased *Thor* mRNA levels, relative to controls, suggesting that insulin signaling was upregulated (Fig. 8b). Next we tested loss- and gain-of-function lines for many glucose sensor candidates such as fructose sensor *Gr43a*^34^, sugar transporter-like *sut3*, anorexia gene *Gr28b*^35^, fat body GPCR *BOSS*^36^, receptors for NPF, sNPF, and dopamine etc. for larval feeding defects. Only larvae lacking expression of *SLC5A11*, a sodium/solute co-transporter–like protein, showed consistently decreased sucrose feeding. Further, *SLC5A11* transcript levels were significantly reduced in all three *Ck1alpha*^*RNAi*^, *Mio*^*RNAi*^, and *Uba1*^*RNAi*^ flyabetic larvae (Fig. 8c, d). A recent study in adult flies, found that *SLC5A11* is used to detect the nutrient value of food, and is expressed in the ellipsoid body of the brain; silencing of *SLC5A11*-expressing neurons effectively decreases feeding^28,37^.

Next we probed if insulin signaling and SLC5A11 may be involved in feeding responses of flyabetic larvae. We found that larvae with reduced expression of key DILPs fed normally on Coomassie-dyed 20% sucrose despite hyperglycemia, while larvae with activated insulin signaling were euglycemic but did not eat (Fig. 8d, Supplementary Fig. 5a). The latter observation was also true for *SLC5A11* mutants that had no detectable dye in their gut despite having normal glucose levels (Fig. 8d). We hypothesized that alleviation of insulin signaling in flyabetic larvae may improve feeding; hence, we cultured *Ck1alpha*^*RNAi*^ and *Mio*^*RNAi*^, and controls, in food containing 100 µM of LY294002, a PI3K inhibitor drug (Cayman Chemicals, #70920), or DMSO. We found that while glucose levels remained high in both treated and untreated flyabetic larvae, they consumed notably more sucrose in the 2nd hour of acute sucrose feeding (Fig. 8e, f, Supplementary Fig. 5b, c). The drug did not alter glucose or feeding in controls. Next, to test if ectopic *SLC5A11* expression in flyabetic larvae can at least partially reverse their feeding defect, we caused hyper-excitation of neuronal SLC5A11 in a *Mio* mutant by expressing a voltage gated bacterial Na + channel, and alternately inhibited K + channel *KCNQ* expression in *Mio* mutants to mimic *SLC5A11* overexpression^38^, using central nervous system (CNS) driver *Elav-Gal4*, respectively. *SLC5A11* expression in *Mio* mutants did not significantly affect glucose levels but improved feeding, detectable after 1 h on dyed 20% sucrose (Fig. 8g, h). Additionally, we attempted and succeeded in a partial rescue of feeding in *Mio*^*RNAi*^ larvae (Supplementary Fig. 5e, f), however the presence of dual drivers (*Dcg-Gal4* and *Elav-Gal4*) in the ‘rescued’ larvae likely contributes to the effects on glucose and feeding.

Finally to begin understanding the interaction of insulin signaling with *SLC5A11* in glucose-mediated feeding, we measured *tobi* and *Thor* mRNA in *SLC5A11* loss-of-function flies, and *SLC5A11* mRNA in flies lacking DILPs. While* tobi* and *Thor* levels remain unchanged in *(Df) SLC5A11* larvae, *SLC5A11* expression was upregulated 6-fold in larvae with reduced DILPs expression but lower by 35% in flies with neuronal activation of insulin signaling, suggesting that *SLC5A11* may lie downstream of insulin signaling (Fig. 8i). Therefore, it appears that *SLC5A11* levels in flies may be regulated by insulin signaling, and SLC5A11 may play a central role in modulating feeding behaviors in response to glucose-sensitive insulin signaling.

### Microarray highlights other feeding behavioral candidates

To identify additional factors that may contribute to glucose sensing, we conducted global microarray screening on *Ck1alpha*^*RNAi*^ and control lines (Supplementary Fig. 6a). These new data underscored the role of SLC5A11 and insulin signaling in diabetic, non-feeding larvae (Table 1). *SLC5A11* was down-regulated by >70% in diabetic, non-feeding *Ck1alpha*^*RNAi*^ larvae once again suggesting its involvement in glucose sensing. Loss of *Ck1alpha* also affected insulin signaling: *dilp6*, antagonist of brain insulins^30–32^, and *ImpL2*, a negative regulator of insulin signaling^39^, were reduced by >80% and >50%, respectively. *Tobi*, induced by insulin^30^, was up-regulated almost 5-fold, supporting our earlier QPCR data indicating elevated insulin signaling in flyabetic larvae.

**Table 1:** Microarray analyses of flyabetic larvae underscored the involvement of insulin signaling and SLC5A11 in glucose-regulated feeding and identified additional candidates

**Gene**	**Ck1alpha** ^**RNAi**^ **signal**	**Control signal**	**Fold-change (ratio)**
Regulation of feeding behavior			
AkhR	174.482	1386.123	0.125877718
SLC5A11	422.7152	1545.414	0.273528776
Pkd2	50.31298	104.9114	0.479575909
smooth	1150.144	2358.59	0.487640497
CCHa1-R	20.50744	42.0979	0.487136888
Poxn	34.13621	69.55724	0.490764297
Gr43a	31.11263	20.174	1.542214236
sNPF	153.9101	97.89566	1.57218512
BM-40-SPARC	8050.446	4991.896	1.612703069
Akh	266.8375	157.3038	1.696319479
DopR2	86.35047	48.18134	1.792197353
5-HT1A	63.78622	35.48811	1.797396931
DAT	1441.932	779.8431	1.849002703
CG10440-RA	357.2795	138.6991	2.57593236
Octbeta2R	65.9241	22.77827	2.894166238
Glucose binding/ homeostasis			
CG8317-RA	1305.513	3325.195	0.392612463
Hexokinase	100.8184	204.147	0.493851979
Sugar transporter-like			
sut3	20.54839	77.10901	0.266484941
CG7342	34.54501	95.08159	0.36331965
CG6356	179.0658	418.4651	0.427910954
CG1208	4131.09	273.7829	15.0889263
Sensory perception of taste			
G-oalpha47A	1330.022	880.7004	1.510186665
Gr85a	37.8274	24.01394	1.575226722
Gr36c	50.23204	30.70763	1.635816245
Gr8a	38.48696	24.14524	1.593977115
Ac78C	637.6902	268.3727	2.376136619
Insulin pathway			
Ilp6	97.32986	574.1712	0.169513657
ImpL2	1454.644	3208.885	0.453317585
tobi	238.7326	49.99505	4.775124737

Rupali Ugrankar et al. show that *Drosophila* larvae with high levels of circulating glucose, but not trehalose, do not eat much. This study suggests that circulating glucose communicates with insulin signaling and sodium/solute co-transporter SLC5A11 in the brain to suppress the larval appetite.

Additionally, the microarray revealed downregulation of *AkhR* (0.13 fold), the receptor of fly glucagon Akh. Expressed in the fat body and gustatory neurons, loss of *AkhR* reduces food intake in flies^29,33,40^. Interestingly, the *CCHamide-1 receptor* (*CCHa1-R*), required for starvation-induced olfactory responses in adult^41^, also showed decreased expression (0.5 fold). The function of *CCHa1-R* and its peptide ligand CCHamide-1 is yet unknown, but its relative CCHamide-2 promotes feeding and release of IPC insulins in larvae^42^. We confirmed lower transcript levels of *AkhR* and *CCHa1-R* in all three flyabetic *Ck1alpha*^*RNAi*^, *Mio*^*RNAi*^, *Uba1*^*RNAi*^ larvae by QPCR (Supplementary Fig. 6b). In addition to the above factors, the microarray revealed moderate changes in a number of genes associated with (mostly adult) fly feeding behaviors, glucose/sugar homeostasis and transport, and taste perception (Table 1). However, feeding was not altered in loss- and gain-of-function lines for several of these genes (e.g. fructose sensor *Gr43a*) individually tested during the screening process. Collectively, our data suggests that insulin signaling and the SLC5A11 sodium/solute co-transporter like protein are key in regulating feeding in flyabetic larvae.

### Feeding screen identifies glucose homeostatic regulators

**Fig. 9:**
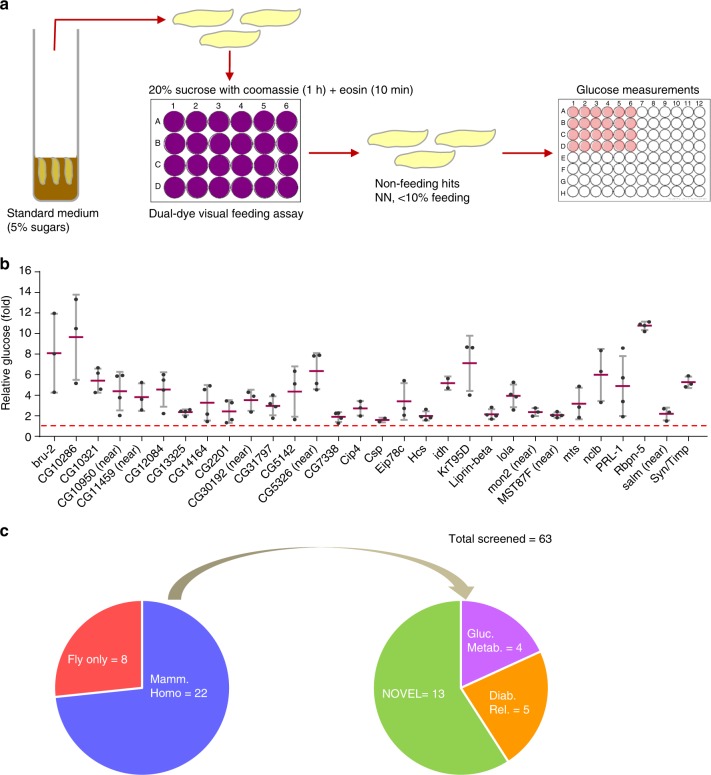
Food aversive mutants identified in sucrose satiety screen are often hyperglycemic. **a** Larvae from >2000 P-element mutant lines were screened for feeding abnormalities using the qualitative dual-dye liquid feeding assay, and those with severe feeding aversion were assayed for glucose. ≥30 larvae per feeding sample. ≥10 larvae per glucose replicate, *n* ≥ 3 each. **b** 30 of 63 P-element “satiety” mutants assayed for glucose levels were found to be hyperglycemic. **c** Of the 30 candidates, the number with mammalian homologs, and glycemia-linked roles and novel genes are indicated. Error bars indicate s.d. Statistical significance was assessed by two-tailed Student’s *t*-test, *P < 0.05, **P < 0.01, ***P < 0.001

To explore the potential of the dual dye qualitative feeding assay to identify satiety and hyperglycemia hits in a high throughput manner, we interrogated 2274 randomly selected P-element mutants (Fig. 9a). We screened for NN larvae (<50% feeding of each dye), and identified 237 (10.4%) hits using these criteria. Among these, we selected 63 lines that fed most poorly (<10% feeding) and quantified their hemolymph glucose levels (Fig. 9a). We found that 30 of the 63 food-averse larvae (~50%) were hyperglycemic (Fig. 9b, Supplementary Table 1); none were significantly hypoglycemic. This is a much higher percentage than we previously observed in our fly glucome screening of randomly selected genes (~9%)^24^, supporting the notion that food averse larvae are enriched in hyperglycemic flyabetes larvae. 22 of the 30 hyperglycemia genes had mammalian homologs, and among these 13 had no known link to glucose regulation in database screening done as previously described^24^ (Fig. 9c, Supplementary Table 1). Together, these data indicate that the fly is a rich source to identify genes that control feeding behavior and those with “satiety” are biased to glucose elevations.

## Discussion

The ability to maintain a relatively constant metabolic milieu is a conserved feature of single and multicellular organisms^10,13,43^. To ensure energy homeostasis, a host of mechanisms, many of which are conserved, have been deployed^38,44–48^. Carbohydrates, such as glucose, are a key source of energy for cells and organisms, and also have important signaling roles^7,38,43,46,47^. Perturbations in glucose availability, consumption, and energy balance due to genetic defects or calorie overload can trigger the onset of metabolic diseases such as type 2 diabetes^2–5,49^. The delineation of roles that carbohydrates and glucose play in metabolic homeostasis, therefore, may have broad implications.

In *Drosophila*, the disaccharide trehalose is present at much higher levels than glucose; many fly interrogations have therefore centered on its metabolic functions^45,50,51^. Recent studies have highlighted glucose as another important regulatory carbohydrate^24,25,52–55^; environmental challenges, excess nutrients, or key genetic mutations can produce significant increases in hemolymph glucose, without concurrent changes in trehalose levels^24,25^. Chronic hyperglycemia can lead to abnormalities in *Drosophila* kidney and cardiac function that resemble aspects of the organ failures in mammalian diabetes^56,57^. *Drosophila* larvae maintain consistent carbohydrate levels despite their relatively continuous feeding activity^24,58^, thus, they represent a distinctive model to uncover key homeostatic mechanisms and to interrogate potential behavioral changes in response to glycemic disturbances.

Feeding behavior is a critical component of energy balance^10,13^. We found that “wild-type” *w*^*1118*^ larvae reared on high sugar (20%) had elevated hemolymph glucose and trehalose, and consumed less food in acute settings compared to counterparts reared on standard media (5% sugars); however, acute exposure to high sucrose was sufficient to increase hemolymph glucose and to decrease feeding rates, though trehalose remained unaffected. These studies indicated that the reduced feeding appeared linked to hemolymph glucose, rather than trehalose, levels. Basal glucose levels in *Drosophila* larvae are relatively low, making detection of hypoglycemia and its effects on baseline feeding rates difficult. Yet, we observed slightly lower glucose levels, unchanged trehalose, but mild upregulation of feeding rates in *w*^*1118*^ larvae chronically cultured in lower (1%) sugar. Next, we examined the feeding behavior of a variety of fly metabolic mutants and flyabetes candidates^24^. Larvae that were averse to consuming sucrose had high glucose levels but variable trehalose levels. Interestingly, in all our screening, we did not isolate larvae with consistently elevated trehalose levels, but normal glucose levels; hypertrehalosemic larvae are almost always hyperglycemic as well. On the other hand, larvae with hyperglycemia can be hypotrehalosemic, eutrehalosemic, or hypertrehalosemic. Thus, glucose elevations, apparently independently of circulating trehalose levels, seem to mediate suppression of sucrose feeding in mid-third instar larvae.

*Drosophila* larvae exert different levels of effort to obtain food that correlate with their level of hunger^16,18^. Larvae transferred to fresh semi-solid media must make a renewed effort to penetrate the food surface and commence eating^16–18^. We noted that markedly hyperglycemic larvae appeared to have diminished motivation to burrow inside low resistance media, taking at least twice as long as controls to initiate foraging, which may indicate abnormal satiety. Such behaviors are quite unusual; typically mid-third instar larvae forage and feed continuously^52,59^. These data are consistent with the possible presence of a sensitive internal cue that detects increases in hemolymph glucose and mediates suppression of foraging and feeding. In support of this notion, we observed that these larvae eventually resume feeding upon restoration of euglycemia. Pharmacological lowering of glucose levels similarly ameliorated feeding behavior. These decisions appear linked to glucose, as we did not observe a correlation between trehalose changes and re-initiation of feeding.

Feeding decisions in flies are guided by palatability and nutritional value of the food, though taste sensing and calorie sensing function independently^48,60,61^. Yet, the presence or absence of carbohydrates or the level of gustatory stimulation of the diet did not appear to influence the feeding behavior of hyperglycemic larvae, supporting the possibility that hyperglycemia may induce a general suppression of feeding. Though, some non-feeding hyperglycemic larvae responded to the presence of yeast, a source of a variety of nutrients and high protein^62,63^. These results indicate the possible presence of systems for the recognition and ingestion of protein, consistent with previous suggestions that protein sensing occurs independently from carbohydrate sensing and that hemolymph amino acid levels may regulate protein feeding through peripheral and internal receptors^63–65^. We also observed increased (wild type) larval feeding on non-nutritive Splenda (sucralose) than sucrose. It has been reported that adult flies display a greater ingestion response (preference index, PI) to agar containing sucralose (PI = 90%) than sucrose (PI = 52%)^66^, suggesting that flies may find sucralose innately more appetitive. Moreover, hungry animals may simply eat more of the super-sweet calorie-free diet to induce satiation. A recent study showed that sucralose increases food intake in flies via a neuronal fasting response comprising the NPF system^67^.

One potential mechanism of carbohydrate sensing may be through induction of expression of insulin-like peptides, DILP2 and DILP3 associated with hemolymph sugar homeostasis^52,68^. Numerous investigations in insects and mammals have indicated a central role of insulin in feeding regulation, possibly through complex interactions with neuromodulators such as NPF/NPY, dopamine, serotonin etc^2,16,18,44,62,65^. In support of these findings, we observed that flyabetic larvae had increased *DILP2* and *DILP3* expression. Thus glucose-mediated suppression of feeding in hyperglycemic larvae may occur, at least in part, via increased insulin activity; altered expression of insulin targets, *tobi* and *Thor* indicated increased peripheral insulin signaling. Further, hyperglycemic larvae deficient in key insulins (DILPs2, 3, 5, and 6) fed normally, while euglycemic larvae with activated insulin pathway ate less. Pharmacological inhibition of insulin signaling by a PI3K inhibitor partially rescued feeding in flyabetic larvae. Our data also support the notion of an internal cue/sensor capable of detecting increases in hemolymph glucose or neuropeptides, and mediating feeding suppression. One such candidate SLC5A11, a member of the sodium/solute co-transporter family, showed lower expression in flyabetic larvae. In flies, *SLC5A11* is upregulated during starvation, and promotes hunger-driven food intake^37^. Neuronal activation of SLC5A11 partially reversed the feeding defect in flyabetic larvae, similar to the effect from suppressing insulin signaling, suggesting its possible involvement and interaction with the insulin pathway in mediating glucose-regulated feeding. QPCR and feeding data suggests that SLC5A11 may function downstream of insulin signaling, and may serve on the pathway of glucose sensing. Sustained hyperglycemia and hyperinsulinemia resulting from loss of key glucose regulatory genes or chronic high sugar diet may induce transcriptional and other irreversible changes. Indeed decrease in *SLC5A11* expression was more pronounced in larvae cultured in 20% sugars relative to those acutely fed a high sucrose diet (Supplementary Fig. 7); this may explain the enhanced severity of the feeding defect in the former versus the latter, despite similar glucose levels (Figs. 1b, c, 2b, c).

Finally, the qualitative dual-dye feeding assay may be useful as an additional high throughput screening tool for satiety and Glucome candidates since feeding defective hits appear to be enriched in glucose regulators. Larvae may alter feeding independently of hemolymph glucose concentrations for other reasons, for example, mutations producing anatomical defects^69,70^, or affecting genes in non-glucose regulated satiety pathways^22,23^, or downstream of glucose sensing. Of note, it is easier and more time- and resource-efficient to first screen for feeding using the dual dye assay followed by glucose quantitation compared to glucose quantitation alone. Thus, the coupled screening approaches are appropriate to find genes important in appetite and glucose homeostatic control, two potential keys to the global epidemics of obesity and diabetes.

In summary, we investigated a potential relationship between circulating carbohydrate levels and feeding behavior in *Drosophila* mid-third instar larvae. We found that high hemolymph glucose levels cause a general, not just sugar-specific, suppression of feeding. Further, hyperglycemic larvae display reduced motivation to work for food, hinting at unusual satiation. Despite its considerable dominance in hemolymph, trehalose levels did not appear to influence larval feeding or foraging. While insulins and SLC5A11 appear to be key players in glucose-responsive feeding, it is quite likely that other cumulative changes, triggered by the hyperglycemia, contribute to the observed feeding defect; in the fly there is substantial and complex crosstalk occurring between the brain and non-IPC insulins, Akh, NPF, sNPF, hugin, drosulfakinin, dopamine etc^33,45,48^. Our microarray data suggests that Akh signaling may be down-regulated in flyabetic larvae. Akh has orexigenic properties, and controls expression of *NPF* and *CCHamide-2*, as well as *DILP2*, *3*, and *6*. This system is likely just one of many as energy balance is critical to organismal survival, and feeding decisions involve additional inputs and complexities^10,12,13^. Fruit flies and hyperglycemic non-feeding larval may offer opportunities to dissect some aspects of feeding behavioral mechanisms, potentially ones which are evolutionarily conserved, that regulate hunger, feeding, and satiety. Appetite control is an important contributor to obesity, and diabetes progression^1,3,6^. Further dissection of the glucose sensing machinery and the role of glucose in appetite signaling cascades may illuminate mechanisms that govern feeding decisions in fly larvae that may also function in mammals.

## Methods

### Fly strains and culture conditions

Flies were reared on standard cornmeal-molasses-yeast-agar food. All *UAS-dsRNA (RNAi)*, P-element, deficiency, and other transgenic strains were obtained from the Bloomington Stock Center. *Dcg-Gal4* flies were described^24^. High sugar media was prepared by adding sucrose (15 g/100 ml) to standard food. This conditioning media also contains a host of other ingredients and caloric sources, for example, yeast and cornmeal. All flies including *RNAi* loss-of-function crosses were maintained at 25 °C. The common laboratory strain *w*^*1118*^ was used as the control line. In experiments involving RNAi, progeny from the crosses between paternal *w*^*1118*^ and maternal *Gal4* driver served as controls. To account for developmental delays, culturing of *w*^*1118*^ larvae on 20% sugars was started 1-2 days before those on 1% and 5% sugars. *Ck1alpha*^*RNAi*^ and *Uba1*^*RNAi*^ cultures were started 1day before *Mio*^*RNAi*^ and controls (*Dcg-Gal4* *>* *w*^*1118*^).

### Larval collection

After egg laying, larvae were cultured in medium to high-density conditions (75-150 larvae/vial) for 6–7 days before harvesting feeding mid-third instars. Vials were rid of older wandering larvae on the sides and food surface. Larvae were collected in 630 µm mesh-fitted baskets (Genesee) and washed to get rid of adherent food particles. Baskets were lowered into a tray of water to allow dead flies to float out, and then into a shallow tray of 2 M NaCl so that larvae float to the top. Larvae were transferred with a paintbrush to Pyrex 9 depression glass spot plates (Corning), and used for different assays.

### Glucose and trehalose assays

Larvae were divided into 4 piles (10–12 larvae each) on a strip of parafilm. Larvae were bled by tearing the cuticle with Dumont 5 forceps (Electron Microscopy Sciences). Two microlitres of colorless hemolymph was aspirated from each pile and separately transferred to 96-well plates (Thermo-Scientific) containing 0.1% N-Phenylthiourea (Sigma-Aldrich) in 50 µl PBS. 150 µl of Autokit Glucose reagent (Wako) was added to each well, and incubated at room temperature for 20 min before measuring absorbance at 505 nm. Glucose concentration was calculated from a standard curve generated with manufacturer’s glucose standards. For trehalose assays, 8 µl of dilute hemolymph was treated with 5 µl of (diluted 8X) porcine kidney trehalase (Sigma) overnight at 37 °C. Ten microlitres of treated sample was assayed for trehalose as described for glucose.

### Liquid feeding assay

Duplicate samples of 30–50 larvae were transferred to 24-well plates containing 250 µl of 20% sucrose + Coomassie blue dye (200 µg/ml). After 50 min, 10 µl of 0.5% Eosin Y (Sigma) fluorescent dye was added to each well. Ten minutes later (total 1 h) larvae were removed and rinsed in water. Through a dissecting scope, larval guts were scored for the presence of Coomassie blue and Eosin dyes using white light and TRITC filter, respectively. The dual dyes allow us to visualize and to quantitate if food consumption might be altered. Larval food consumption was scored as follows: “Yes” (Y) if > 90% of larvae contained dye at levels approximating *w*^*1118*^ controls; “Partial” (P) if between 50–90% of larval guts had any dye, and/or > 90% larvae had dye but at very low levels; “No” (N) if < 50% of larvae had eaten.

### Soft agar response assay

Soft agar food was prepared with 5% sucrose, 10% yeast, and 0.5% or 0.2% agar, otherwise nutritionally similar to standard fly media Agar was melted in the microwave and Coomassie dye (200 µg/ml) was added to allow easy visualization of the whitish-yellow-hued larvae, and quantification of food ingestion. Agar was poured into 5 cm Petri plates to form a ~5 mm thick layer. Once dry, 100 larvae were introduced to the center of the agar plate and monitored for foraging activity.

### Insoluble protein feeding assay

150 larvae were transferred to 24-well plates containing 400 µl of 5% yeast extract solution. Addition of Coomassie dye, prepared in water instead of ethanol, to 5% yeast extract produces insoluble blue dye-protein complexes. After 2 h, larvae were removed, rinsed in water and then methanol. Both wells and larvae were photographed to record dye-protein ingestion.

### Food ingestion quantification

Larvae fed Coomassie dye-supplemented media were harvested, washed in water and methanol, blotted dry, and transferred to 1.5 ml Eppendorf tubes on ice (30 larvae/sample, in triplicate). Larvae were homogenized in 200 µl of 100% methanol. Samples were centrifuged at 14,000 rpm for 10 min; 175 µl of the supernatant was transferred to fresh tubes containing 175 µl of dH_2_O, and centrifuged again. Absorbance of this supernatant was measured at 595 nm, and dye amount (food ingested) was calculated by comparing with a standard curve generated from a range of Coomassie dye concentrations.

### Quantitative RT-PCR and microarray

RNA for Quantitative RT-PCR was extracted from mid-third instar feeding larvae using Trizol Reagent (Ambion Life Technologies) as per manufacturer’s instructions. cDNA was synthesized from 1 µg of RNA using ReadyScript™ cDNA Synthesis Mix (Sigma). QPCR was performed on the BioRad CFX96 Real-Time System; data was normalized to endogenous control rp49. RNA for Microarray was extracted from larvae using RNA extraction kit (Qiagen), and sent to the UTSW Genomics and Microarray Core Facility for microarray analyses by Affymetrix GeneChip Array. Primer sequences for all transcripts amplified are listed in Supplementary Table 2.

### Statistical analysis

Error bars indicate standard deviation (s.d.). Statistical significance was assessed by two-tailed Student’s *t*-test, *P < 0.05, **P < 0.01, ***P < 0.001.

### Data Availability

The microarray dataset generated during the current study are available in the GEO repository (GSE116003). All other relevant data is available from the authors on request.

## Electronic supplementary material

Supplementary Information
