# Supplementary material for: Circulating glucose levels inversely correlate with Drosophila larval feeding through insulin signaling and SLC5A11
Source: Commun Biol. 2018 Aug 13;1:110. doi: 10.1038/s42003-018-0109-4 (PMC6123810; doi:10.1038/s42003-018-0109-4)
Supplement: Supplementary file 1 — Supplementary Information [file 42003_2018_109_MOESM1_ESM.pdf]

Supplementary Information

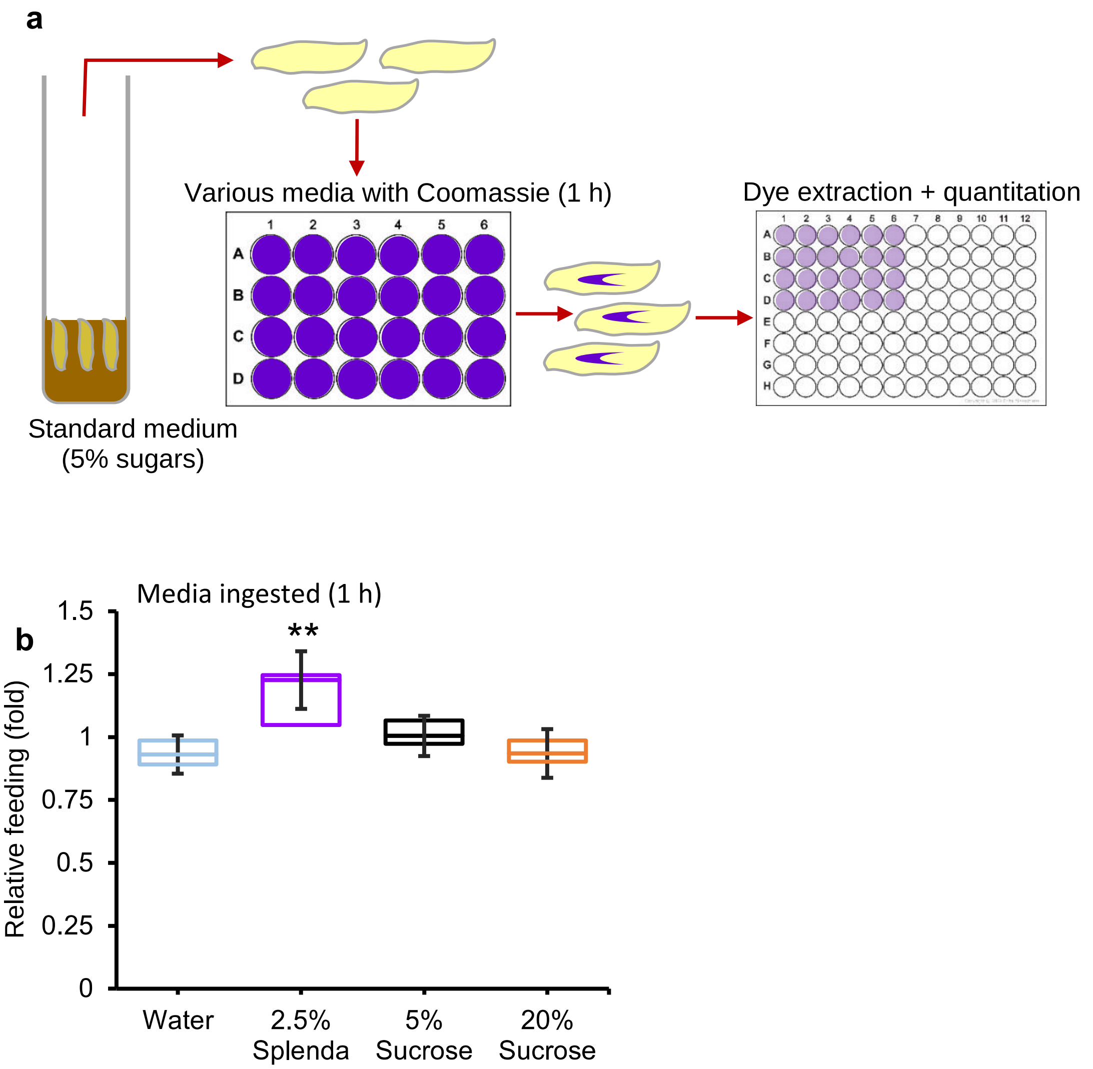

Supplementary Figure 1: *w*^{1118} controls ingest more Splenda than water and sucrose

(a-b) *w*^{1118} larvae reared on standard food were transferred to water, 2.5% Splenda, 5% sucrose, or 20% sucrose supplemented with Coomassie dye for 1 h (a), and then dye ingested was quantitated (b) n = 8 each (20 larvae per replicate). Error bars indicate s.d. Statistical significance was assessed by two-tailed Student's t-test, *P < 0.05, **P < 0.01, ***P < 0.001.

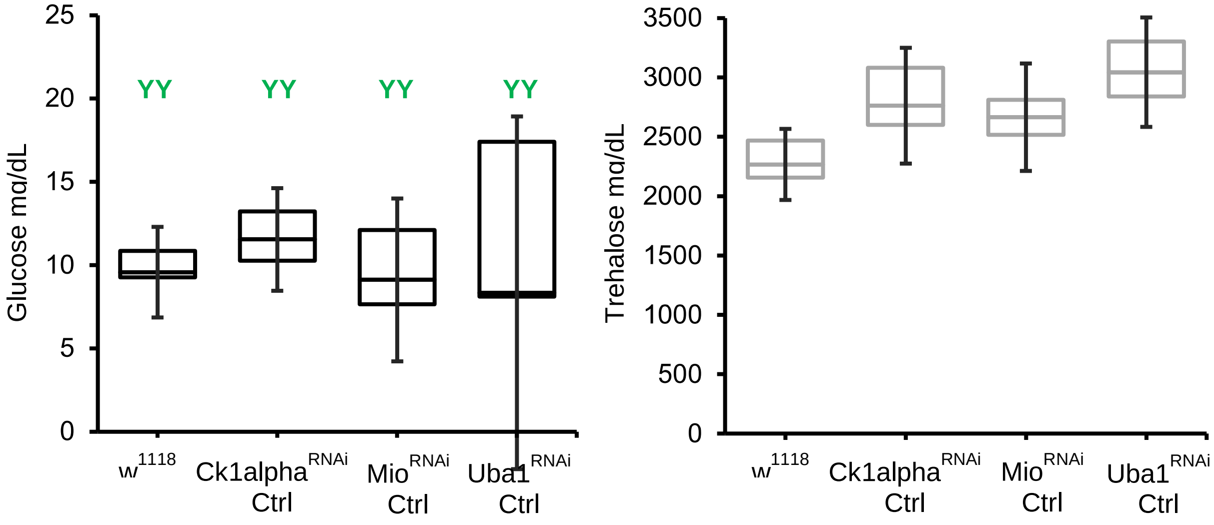

Supplementary Figure 2: Glucose and trehalose levels measured in un-induced UAS-RNAi larvae, relative to Dcg-Gal4>w^{1118} controls

n ≥ 3 each (20 larvae per replicate). Error bars indicate s.d. Statistical significance was assessed by two-tailed Student's t-test, *P < 0.05, **P < 0.01, ***P < 0.001.

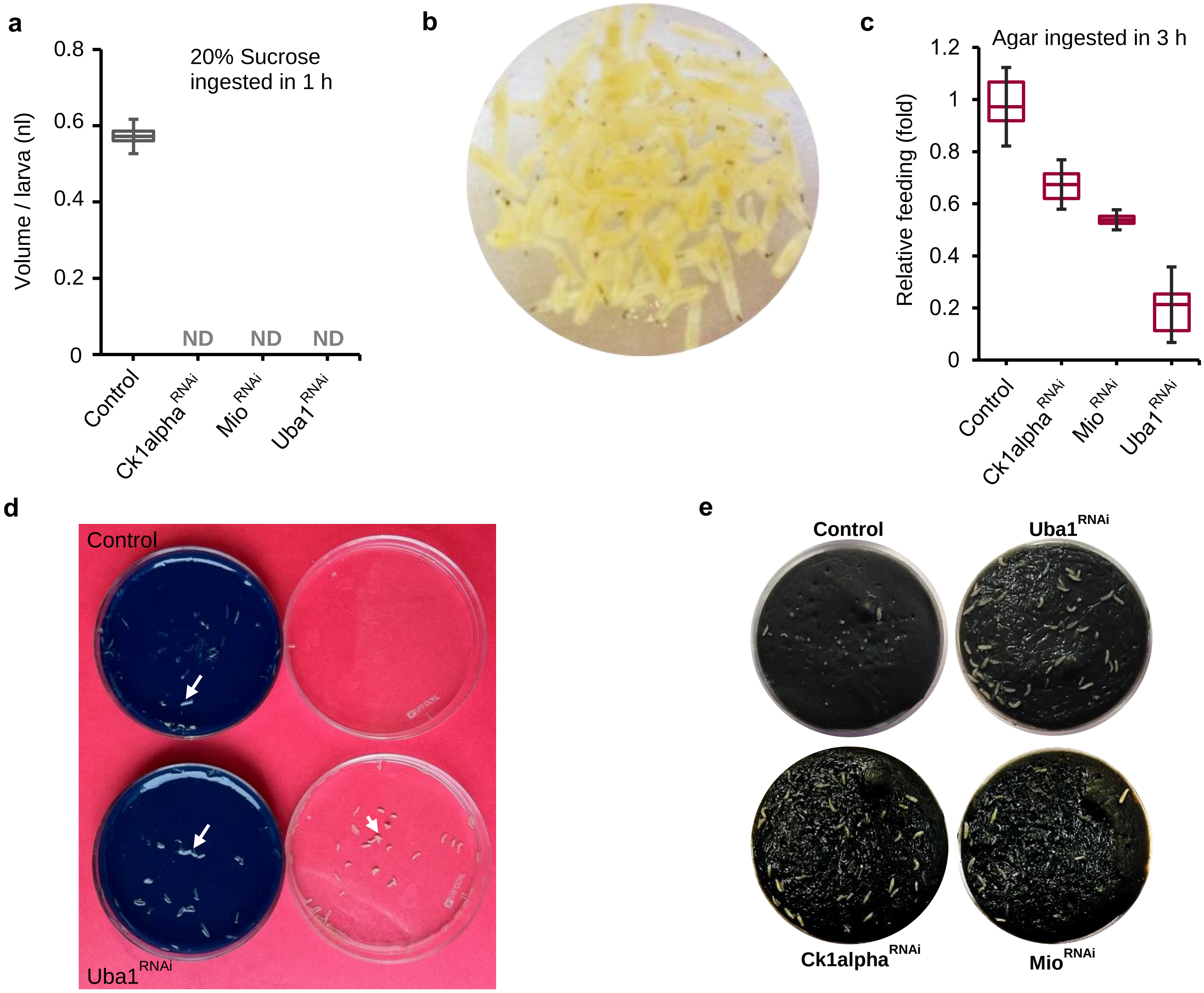

Supplementary Figure 3: Hyperglycemic non-feeding larvae delay foraging in low-resistance media

(a) *w*^{1118}; *Dcg-Gal4* control and “flyabetic” larvae were provided Coomassie dye-supplemented 20% sucrose and after 1 h ingested dye was quantitated. n = 6 (20 larvae per replicate) (b) *w*^{1118}; *Dcg-Gal4* control larvae provided 20% sucrose without dye were imaged after 1 h. (c) Control and flyabetic larvae were transferred to 0.5% agar food

plates containing Coomassie dye, and agar ingested was quantitated after 3 h. $n = 3$ (20 larvae per replicate). (d and e) Control and flyabetic larvae ($n = 100$ each) were transferred to the center of Coomassie-dyed 0.2% lower agar food plates (d) and soft yeast plates (e), and foraging initiative was monitored. Images were taken at 2 h (d) and 1 h (e) after transfer.

Error bars indicate s.d. Statistical significance was assessed by two-tailed Student’s t-test, *$P < 0.05$, **$P < 0.01$, ***$P < 0.001$.

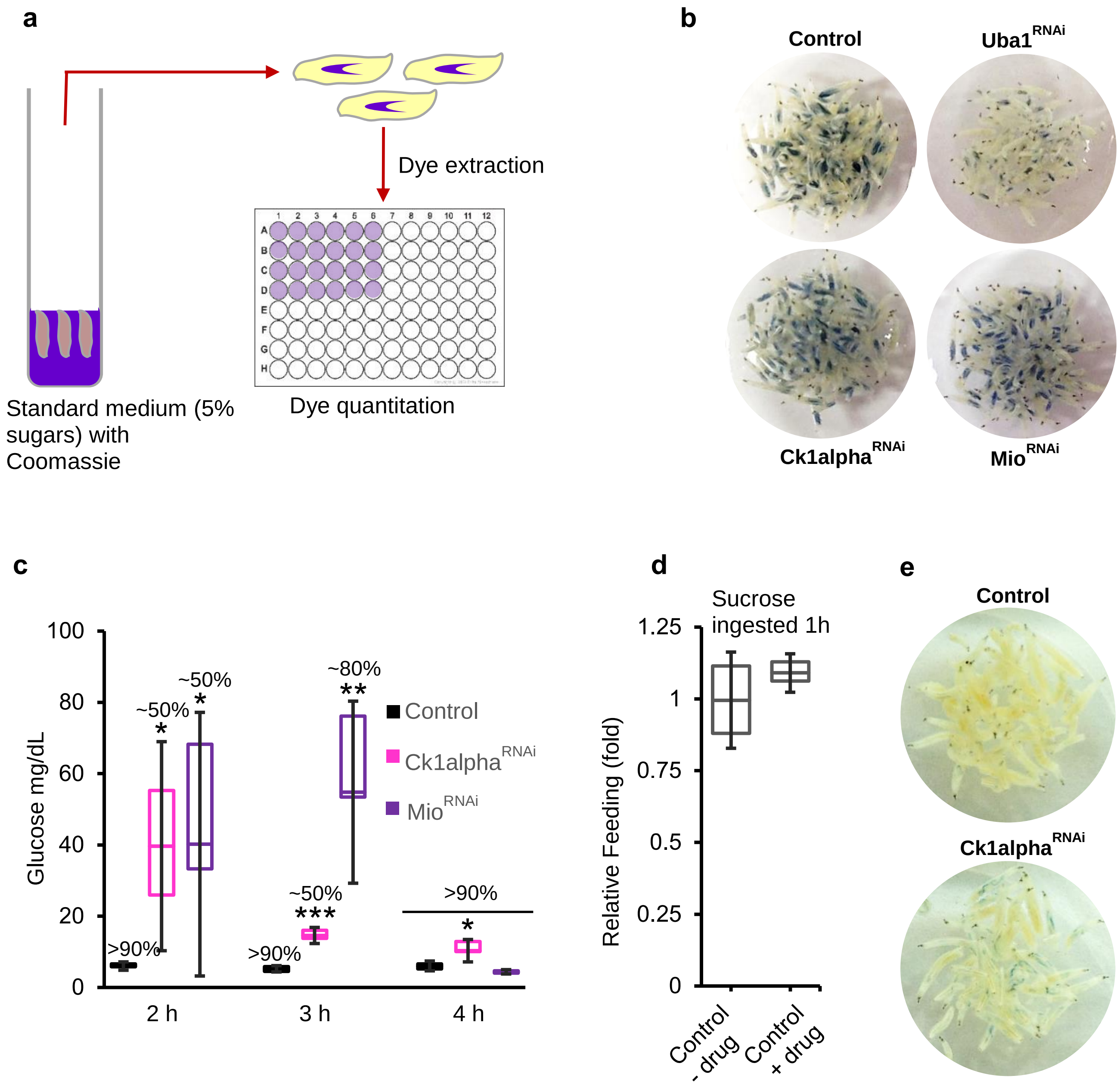

Supplementary Figure 4: Hyperglycemic larvae ingest their rearing media

(a-b) Control and flyabetic larvae reared on standard food supplemented with Coomassie dye (a) are harvested and photographed (b). (c) Glucose and feeding was tracked in control and flyabetic larvae (≥ 200 larvae of each) at 2 h, 3 h, and 4 h post-transfer to 5% sucrose. For glucose measurements, n ≥ 3 each (≥ 10 larvae per

replicate), ≥ Feeding assessed in ≥50 larvae per sample at each time point. (d) Relative amounts of Coomassie-dyed 20% sucrose ingested in 1 h by control larvae cultured in standard food, in the absence or presence of drug Phlorizin. n ≥ 3 each (20 larvae per replicate) (e) Flyabetic *Ck1alpha*^{RNAi} larvae cultured in standard food, with or without Phlorizin, were provided Coomassie-dyed 20% sucrose for 1 h, then harvested and imaged.

Error bars indicate s.d. Statistical significance was assessed by two-tailed Student's t-test, *P < 0.05, **P < 0.01, ***P < 0.001.

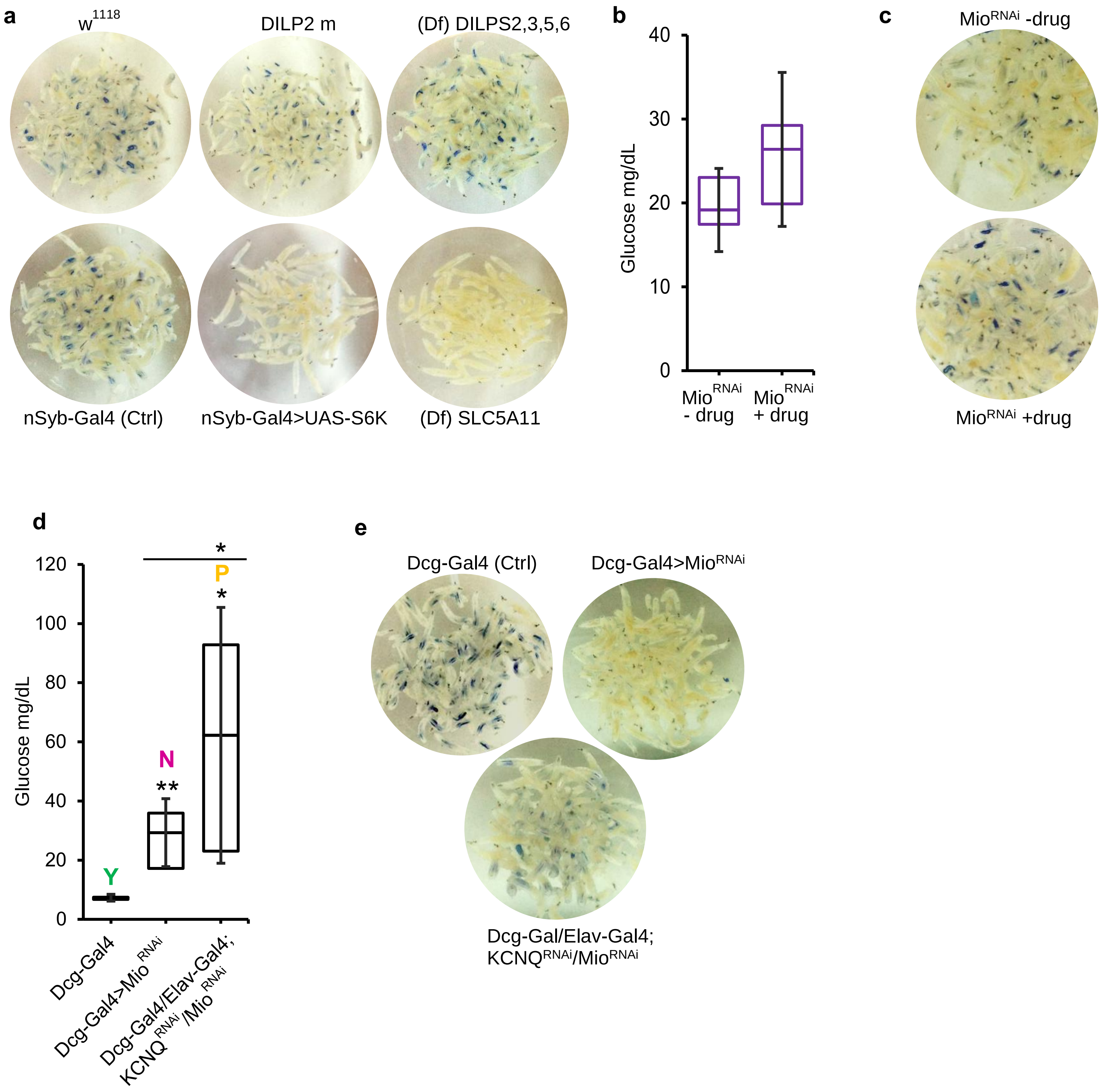

Supplementary Figure 5: Insulin signaling and SLC5A11 may regulate glucose-responsive feeding behavior.

(a) Feeding on Coomassie-dyed 20% sucrose was compared in larvae with altered insulins/ insulin signaling, and SLC5A11, relative to controls. (b-c) Glucose and feeding were assayed in Mio^{RNAi} flyabetic larvae reared in the absence or presence of a PI3K-inhibitor drug. n = 10 each (≥ 10 larvae per glucose replicate) (d-e) Glucose and feeding were compared in Mio^{RNAi} larvae with enhanced excitation of neuronal SLC5A11, relative to Mio^{RNAi} flyabetic larvae. n ≥ 4 (≥ 10 larvae per glucose replicate)

Feeding experiments used ≥ 30 larvae per treatment. Error bars indicate s.d. Statistical significance was assessed by two-tailed Student’s t-test, *P < 0.05, **P < 0.01, ***P < 0.001.

a

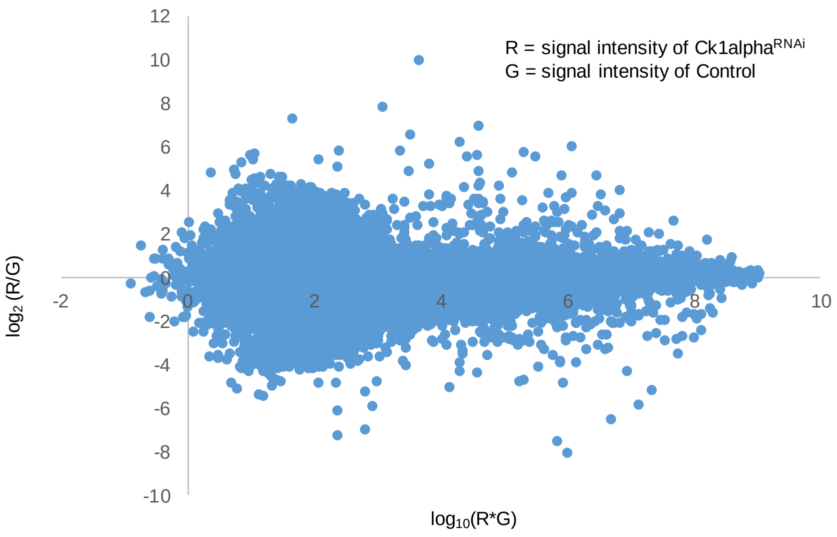

b

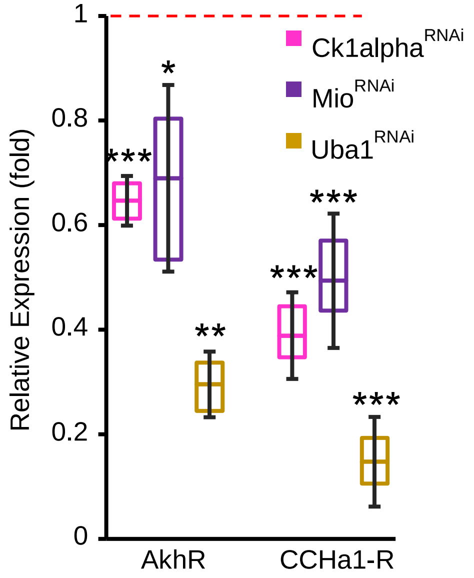

Supplementary Figure 6: Microarray analyses of flyabetic larvae revealed additional feeding behavioral candidates

(a) Ratio-Intensity scatter plot of raw microarray data.

(b) Transcript levels of *AkhR* and *CCHa1-R* in *Ck1alpha^{RNAi}, Mio^{RNAi}, and Uba1^{RNAi}* flyabetic larvae relative to controls, detected by QPCR. n = 3 each (20 larvae per replicate).

Error bars indicate s.d. Statistical significance was assessed by two-tailed Student's t-test, *P < 0.05, **P < 0.01, ***P < 0.001.

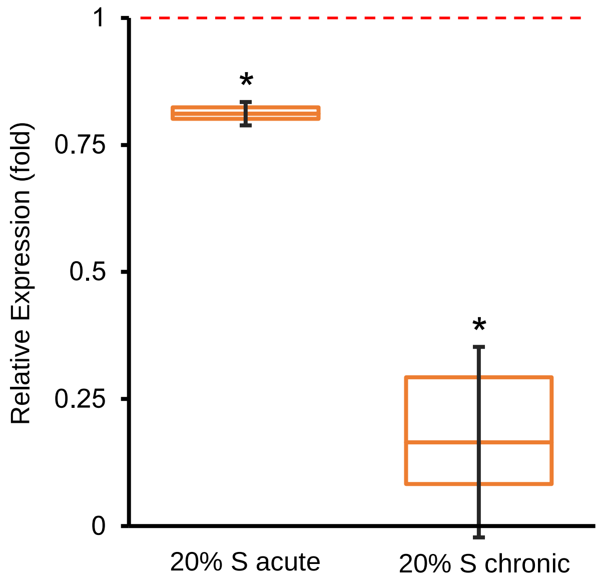

Supplementary Figure 7: *SLC5A11* expression is more severely reduced in *w*[1118] larvae chronically fed a 20% high sucrose diet relative to those exposed to 20% sucrose for only 4 h.

n ≥ 3 each (20 larvae per replicate). Error bars indicate s.d. Statistical significance was assessed by two-tailed Student's t-test, *P < 0.05, **P < 0.01, ***P < 0.001.

Gene/Region Disrupted	Stock#	Mammalian homolog	Molecular/Biological Function	Gluc. Metab. /Diab Rel.
bru-2	22296	Celf3,5,6	nucleotide binding	No
CG10286	21354	Heatr3	unknown	No
CG10321	22343	n/a	DNA binding	No
CG10950 (near)	23179	n/a	protein transmembrane transporter	No
CG11459 (near)	20785	n/a	proteolysis	No
CG12084	23756	Zer1	ubiquitin-protein transferase	No
CG13325	24195	n/a	acyltransferase 3	No
CG14164	22384	Lsm12	unknown	No
CG2201	24891	Chkb	choline kinase	Yes
CG30192 (near)	23218	n/a	unknown	No
CG31797	19630	n/a	unknown	No
CG5142	24191	Ttc30a1,a2,b	transporter	No
CG5326 (near)	20947	ELOVL1,4,7	fatty acid elongation	Yes
CG7338	21167	Tsr1	ribosome biogenesis	No
Cip4	20291	CIP4	phospholipid binding	Yes
Csp	22563	Dnajc5	exocytosis	Yes
Eip78C	23386	Nr1D1,2	steroid hormone receptor	Yes
Hcs	20945	Hlcs	biotin protein ligase	No
idh	20183	IDH1,2	(NADP+) activity	Yes
KrT95D	20754	Pacs1,2	protein targeting to golgi	No
Liprin-beta	22658	Liprin-beta2	protein binding	No
lola	23202	ZBTB20	nucleic acid binding	Yes
mon2 (near)	21161	Mon2	lipid metabolic process	No
MST87F (near)	21137	n/a	spermatogenesis	No
mts	21421	Ppp4c,6c,2ca,2cb	cell cycle	Yes
nclb	20206	PWP1	chromatin DNA binding	No
PRL-1	20971	Ptp4a1,2,3	phosphatase, mitosis	No
Rbpn-5	22472	Rabep1,2	Rab GTPase binding	No
salm (near)	23760	n/a	zinc finger, nucleic acid binding	No
Syn/Timp	23762	Syn1,2,3/Timp1,2,3,4	ATP binding	Yes

Supplementary Table 1: Hyperglycemia hits from the sucrose aversion screen

30 feeding defective P-element lines were identified as hyperglycemia candidates. Mammalian homolog, molecular/biological function, and involvement in glucose-related role are also listed.

	Primer Sequences
DILP2 F	TCTGCAGTGAAAAGCTCAACGA
DILP2 R	TCGGCACCGGGCATG
DILP3 F	AGAGAACTTTGGACCCCGTGAA
DILP3 R	TGAACCGAACTATCACTCAACAGTCT
DILP6 F	TCGGTTACGTTCTGCAAGTC
DILP6 R	CACGGAATACGAACACAGACG
tobi F	TGATGTCTTCGCCCAAAAGG
tobi R	CCATGCCACCTATACACCTTAC
Thor F	CATGCAGCAACTGCCAAATC
Thor R	CCGAGAGAACAAACAAGGTGG
SLC5A11 F	CTGGGCCAATACTGTAGGCA
SLC5A11 R	GGACAGTCGGTACAGTGGAA
AkhR F	ATGATTCCTGTCTCAGTGCG
AkhR R	CCATTTGCTCCATGTGATGTC
CCHa1-R F	GACCGGAAACCTACATCGTG
CCHa1-R R	CCAGGGCCAGTGATAAAATG
rp49 F	CGGATCGATATGCTAAGCTGT
rp49 R	GCGCTTGTTCGATCCGTA

Supplementary Table 2: Primer sequences of all mRNAs detected by quantitative PCR
